# Breath-held high-resolution cardiac T_2_ mapping with SKRATCH

**DOI:** 10.1186/1532-429X-18-S1-P27

**Published:** 2016-01-27

**Authors:** Emeline Lugand, Jérôme Yerly, Hélène Feliciano, Jérôme Chaptinel, Matthias Stuber, Ruud B van Heeswijk

**Affiliations:** 1 University Hospital (CHUV) and University of Lausanne (UNIL), Lausanne, Switzerland; 2 Center for Biomedical Imaging (CIBM), Lausanne and Geneva, Switzerland

## Background

**Figure 1:**
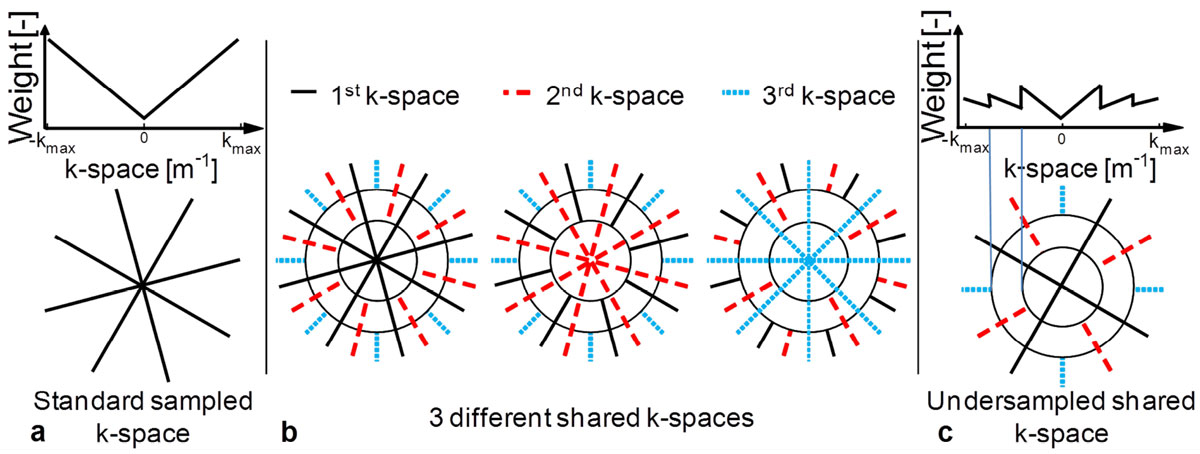
**Schematic overview of the KWIC filter**. **a.** A radial k-space sampling pattern shown below its DCF along one radial line. The DCF is used to weigh the k-space points. **b.** Three similar k-spaces that share their periphery through the KWIC filter, thus increasing the local sampling density and decreasing the local weight attributed by the DCF. The radii outside of which data were added were defined through the Nyquist criterion. **c.** An undersampled KWIC-filtered k-space. While the number of lines has decreased, the periphery of k-space still has a higher sampling density than the standard radial k-space in a.

Several cardiac T_2_ mapping techniques with varying T_2_ preparation (T_2_Prep) times have been proposed for the quantification of cardiac edema [1–3]. Among these, radial T_2_ mapping, which is robust to motion artifacts, suffers from a low signal-to-noise ratio (SNR) caused by the undersampling of the k-space periphery and by its density compensation function (DCF) (Fig. 1a). However, since the contrast of an image is mainly determined by the center of its k-space, the T_2_-weighted images can share their k-space periphery using the KWIC (K-space Weighted Image Contrast) filter (Fig. 1b) to reduce undersampling artifacts [4]. This allows for higher undersampling (Fig. 1c) and thus for a decrease in acquisition time [5].

We demonstrated that navigator-gated KWIC-filtered cardiac T_2_ mapping (Shared K-space RAdial T_2_ Characterization of the Heart, SKRATCH) enables a considerable decrease in acquisition time while maintaining the T_2_ precision [5]. The goal of this study was to extend this approach to a short breath-held high-resolution T_2_ map acquisition and to compare its performance to navigator-gated T_2_ mapping.

## Methods

The novel breath-held SKRATCH protocol consisted of a GRE sequence with a continuously increasing golden-angle radial acquisition. This ensured a unique k-space trajectory for all 64 lines of each of the 4 T_2_Prep durations (0/30/45/60 ms), pixel size of 1.2 × 1.2 × 8 mm^3^ and a total duration of 7 heartbeats. As reference, a navigator-gated radial cardiac T_2_ mapping GRE sequence was acquired with 3 T_2_Prep durations (0/30/60 ms), 308 lines/image and a pixel size of 1.25 × 1.25 × 5 mm^3^ [3]. Images were acquired at 3T (Magnetom Prisma, Siemens Healthcare) in 17 healthy volunteers at the same midventricular short-axis orientation with both protocols. The T_2_ maps were segmented according to the AHA guidelines [6]. The mean T_2_ value (μ_T2_) and the relative standard deviation (σ_R_ = standard deviation/ μ_T2_) of each segment as well as the myocardial area were calculated and tested for significant differences. The SKRATCH T_2_ map was acquired twice in 11 of the volunteers for Bland-Altman reproducibility analysis.

## Results

**Figure 2:**
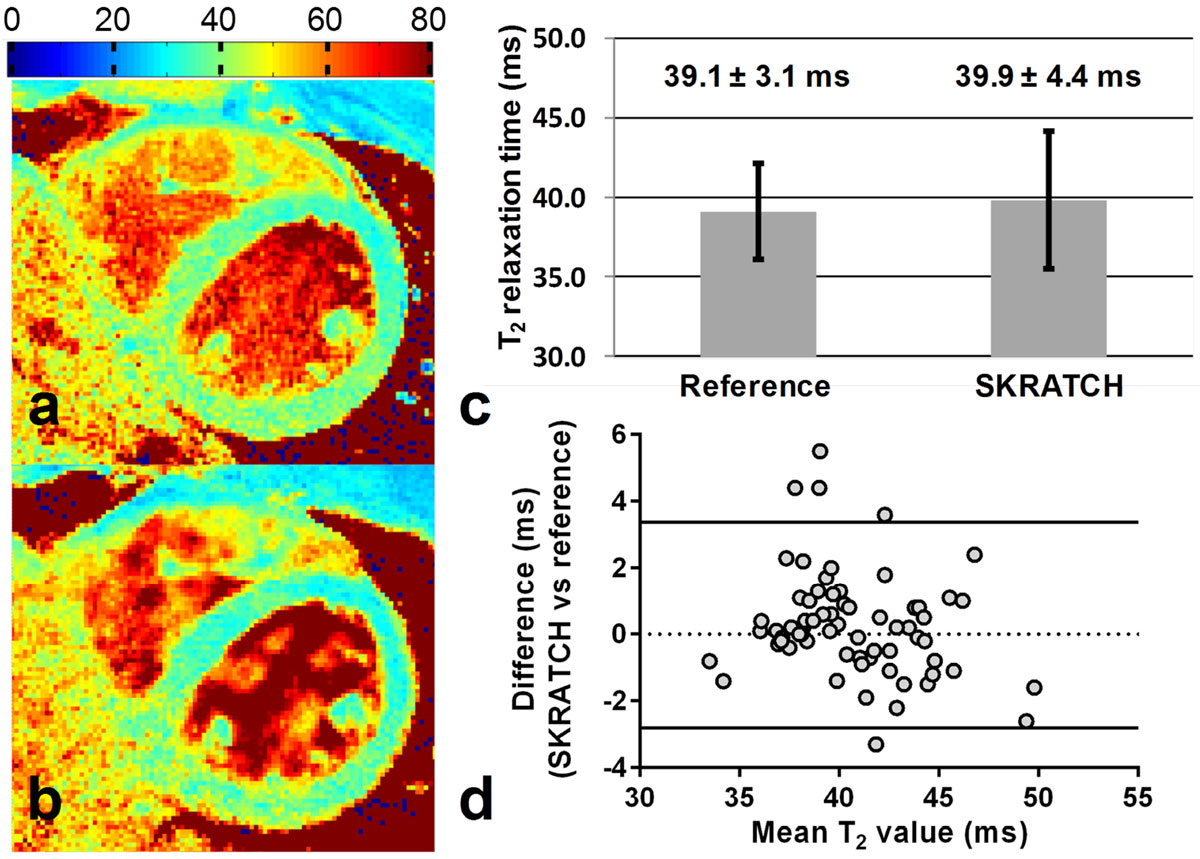
**A comparison of navigator-gated and breath-held high-resolution T**_**2**_ **maps in healthy volunteers**. **a,b**. The standard navigator-gated T_2_ map and breath-held SKRATCH T_2_ map respectively. Note that the maps are homogeneous and have similar myocardial surface available for analysis. The color bar indicates the T_2_ relaxation time in ms. **c**. The mean T_2_ values and standard deviations of the 17 healthy volunteers show a slight increase in standard deviation for the breath-held SKRATCH acquisition. **d**. The Bland-Altman analysis of the difference in mean T_2_ values for 11 volunteers. The dotted line represents the mean with a bias of 0.28, while the continuous lines represent the 95% confidence interval (1.96 × standard deviation).

The SKRATCH T_2_ maps had average values of 39.9 ± 4.4 ms, while those of the reference T_2_ maps were 39.1 ± 3.1 ms (p = 0.04, Fig. 2a-c). σ_R_ increased from 8 ± 2% for the standard T_2_ maps to 11 ± 2% for the SKRATCH T_2_ maps (p < 0.001). The myocardial area decreased from 643 ± 155 to 585 ± 121 pixels for the SKRATCH T_2_ maps (a 10% decrease, p = 0.008). The repeatability analysis resulted in a confidence interval of ± 3.09 ms (Fig. 2d).

## Conclusions

The SKRATCH T_2_ maps were highly similar to the reference high-resolution T_2_ maps, while the shortening to breath-hold duration came at the cost of an acceptably small increase in standard deviation and decrease in myocardial area. These encouraging results will need to be validated in future high-resolution studies in patients.
